# Supplementary material for: Computational and Experimental Analysis of Gold Nanorods in Terms of Their Morphology: Spectral Absorption and Local Field Enhancement
Source: Nanomaterials (Basel). 2021 Jun 28;11(7):1696. doi: 10.3390/nano11071696 (PMC8308185; doi:10.3390/nano11071696)
Supplement: Supplementary file 1 [file nanomaterials-11-01696-s001.zip › nanomaterials-1238247-supplementary.pdf]

Supplementary Information

The nanorods analyzed in this paper are made of gold and are small compared with *l*. Their metallic character induces a strong plasmonic resonance that generates a large absorption. Therefore, the scattering contribution is much smaller than the contribution from absorption mechanisms. Then, for the case of AuNRs, the total extinction cross section, $s_{ext} = s_{abs} + s_{sca}$, is mostly determined by s_{abs}. Fig. S1 shows the spectral dependence of s_{ext}, s_{abs}, and s_{sca} for a given realization of our AuNRs (d_l = 52 nm, d_t = 17 nm, and $q = 0_o$). We can see how the extinction cross-section and the absorption cross-section have similar shapes and the spectral locations of the maxima are almost unchanged.

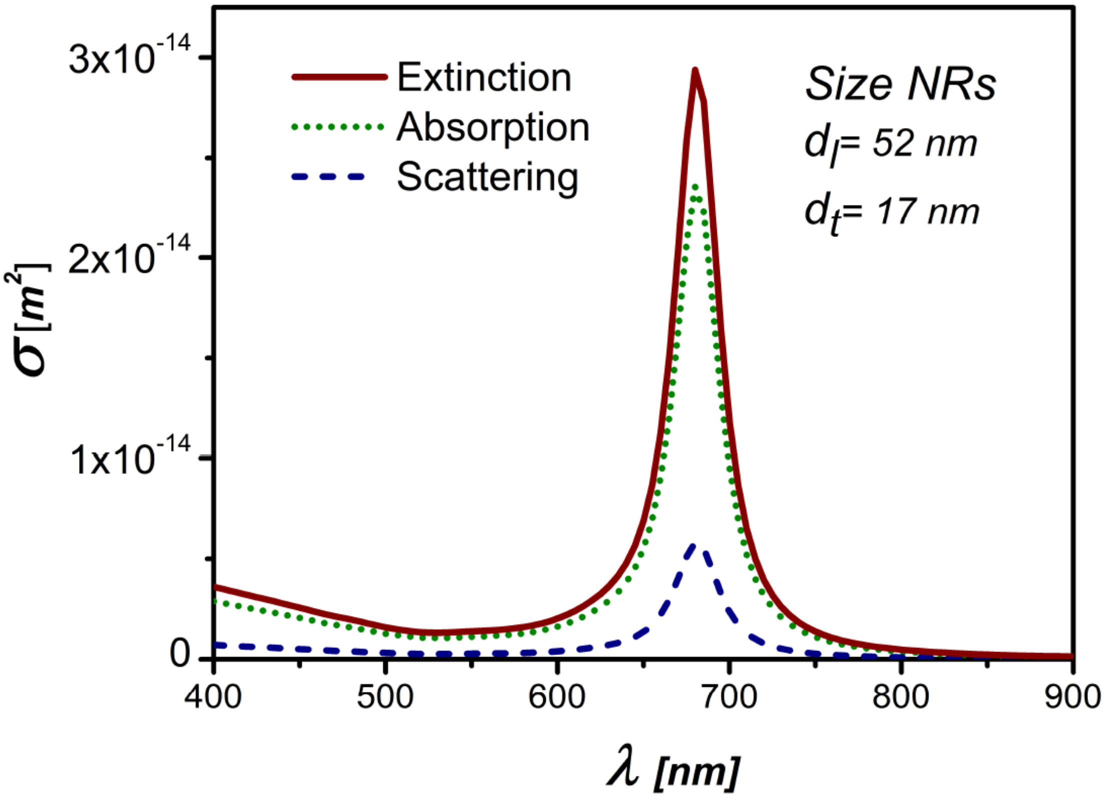

Figure S1: Spectral cross section for extinction, absorption and scattering. The plots are for a nanorod with geometric parameters d_l = 52 nm and d_t = 17 nm, for a polarization along the long axis of the nanorod.
